# Regulators of G-Protein signaling RGS10 and RGS17 regulate chemoresistance in ovarian cancer cells

**DOI:** 10.1186/1476-4598-9-289

**Published:** 2010-11-02

**Authors:** Shelley B Hooks, Phillip Callihan, Molly K Altman, Jillian H Hurst, Mourad W Ali, Mandi M Murph

**Affiliations:** 1 Department of Pharmaceutical and Biomedical Sciences, The University of Georgia, Athens, GA, USA

## Abstract

**Background:**

A critical therapeutic challenge in epithelial ovarian carcinoma is the development of chemoresistance among tumor cells following exposure to first line chemotherapeutics. The molecular and genetic changes that drive the development of chemoresistance are unknown, and this lack of mechanistic insight is a major obstacle in preventing and predicting the occurrence of refractory disease. We have recently shown that Regulators of G-protein Signaling (RGS) proteins negatively regulate signaling by lysophosphatidic acid (LPA), a growth factor elevated in malignant ascites fluid that triggers oncogenic growth and survival signaling in ovarian cancer cells. The goal of this study was to determine the role of RGS protein expression in ovarian cancer chemoresistance.

**Results:**

In this study, we find that RGS2, RGS5, RGS10 and RGS17 transcripts are expressed at significantly lower levels in cells resistant to chemotherapy compared with parental, chemo-sensitive cells in gene expression datasets of multiple models of chemoresistance. Further, exposure of SKOV-3 cells to cytotoxic chemotherapy causes acute, persistent downregulation of RGS10 and RGS17 transcript expression. Direct inhibition of RGS10 or RGS17 expression using siRNA knock-down significantly reduces chemotherapy-induced cell toxicity. The effects of cisplatin, vincristine, and docetaxel are inhibited following RGS10 and RGS17 knock-down in cell viability assays and phosphatidyl serine externalization assays in SKOV-3 cells and MDR-HeyA8 cells. We further show that AKT activation is higher following RGS10 knock-down and RGS 10 and RGS17 overexpression blocked LPA mediated activation of AKT, suggesting that RGS proteins may blunt AKT survival pathways.

**Conclusions:**

Taken together, our data suggest that chemotherapy exposure triggers loss of RGS10 and RGS17 expression in ovarian cancer cells, and that loss of expression contributes to the development of chemoresistance, possibly through amplification of endogenous AKT signals. Our results establish RGS10 and RGS17 as novel regulators of cell survival and chemoresistance in ovarian cancer cells and suggest that their reduced expression may be diagnostic of chemoresistance.

## Background

Ovarian cancer is a solid malignancy often initially responsive to cytotoxic chemotherapy, but the high 5-year mortality rate (>55%) is a result in part of the tumor cells' ability to develop resistance to chemotherapy. This "chemoresistance" is the specific acquired resistance against the action of therapeutic agents (i.e. cytotoxic chemotherapy) that makes further treatment refractory. Its occurrence in patients with ovarian cancer is unfortunately common because this disease has characteristically dormant cells within the peritoneal cavity that proliferate slowly, allowing for the development or selection of resistance to aggressive treatment. Therefore it is this pool of persistent, dormant, chemoresistant cells that prevents the clinical and pharmacological ability to cure ovarian cancer [1]. The molecular and genetic changes that drive the development of chemoresistance are unknown, and this lack of knowledge is a major obstacle in preventing and predicting clinical chemoresistance.

Lysophosphatidic acid (LPA), an established mediator of ovarian cancer cell growth and survival [2,3], is abundantly present in the tumor microenvironment [4-7], suggesting that amplification of autocrine or paracrine LPA-mediated survival signaling could play a role in chemoresistance. LPA functions by activating a family of G-protein coupled receptors (GPCRs), which activate heterotrimeric G-proteins to couple extracellular LPA to activation of cellular signaling cascades such as MAP kinase growth pathways and AKT survival pathways [8]. Like all GPCRs, LPA receptors trigger cellular responses by stimulating the transition of G-proteins from their inactive GDP-bound form to the active GTP-bound form. Thus, G-proteins are the critical mediators of LPA signaling cascades, and proteins that regulate G-protein activity control the strength of LPA stimulated responses in ovarian cancer cells.

A critical regulatory point in the G-protein activity cycle is the deactivation of G-proteins by GTP hydrolysis, a step which is markedly enhanced in cells by GTPase activating proteins (GAPs). Regulators of G-protein Signaling (RGS) proteins are a highly diverse family of proteins that function as GAPs to accelerate the deactivation of heterotrimeric G-proteins, thus terminating signaling initiated by GPCRs [9,10]. We have recently shown that RGS proteins suppress LPA stimulated growth signals in ovarian cancer cells [8,11], and identified over a dozen RGS transcripts expressed in ovarian cancer cells, many of which are differentially expressed in normal ovarian cells versus ovarian cancer cell lines [12]. Similarly, comparing RGS expression in normal versus tumor tissue using existing databases reveals significant changes in RGS expression during ovarian cancer progression, suggesting RGS expression levels are altered as ovarian epithelial cells undergo oncogenic transformation [13].

Taken together, the ability of LPA to function as a survival factor in ovarian cancer cells, the ability of RGS proteins to suppress LPA stimulated G-protein activity, and the differential expression of RGS proteins in ovarian cancer cells suggest that changes in RGS expression levels during ovarian cancer progression may regulate the strength of LPA mediated survival signals and contribute to chemoresistance. We hypothesized that if expression of RGS proteins is decreased in ovarian cancer cells, this may amplify tonic constitutive LPA-stimulated cell survival pathways, allowing cells to overcome cytotoxicity. Indeed, we report here that gene expression microarray analysis comparing chemotherapy-sensitive and chemoresistant ovarian cancer cells reveals downregulation of specific RGS transcripts during acquired chemoresistance. We further demonstrate that RGS10 and RGS17 expression regulates the cellular toxicity of multiple cytotoxic chemotherapeutics, which ultimately enhances the viability of these tumor cells in the presence of chemotherapy. Finally, we observed that RGS10 and RGS17 are able to suppress LPA-induced activation of the survival factor AKT, suggesting a mechanistic model for RGS10 and RGS17 control of chemoresistance in ovarian cancer cells.

## Methods

### Cells and Reagents

We purchased SKOV-3 cells from American Type Culture Collection (Manassas, VA). These cells were maintained in McCoy's 5A medium (Mediatech, Inc., Manassas, VA.) supplemented with 10% FBS (PAA Laboratories, Inc., Etobicoke Ontario, Candada). Both the parental and the MDR-HeyA8 cell line, a taxane-resistant line generated by the long-term exposure to paclitaxel, were kind gifts from Dr. Isaiah J. Fidler (Department of Cancer Biology, University of Texas M.D. Anderson Cancer Center, Houston, TX) and are presented elsewhere [14]. MDR-HeyA8 cells are maintained in RPMI 1640 medium with 300 ng/mL paclitaxel with 15% FBS and HeyA8 cells are maintained in RPMI medium with 15% FBS. Lysophosphatidic acid (18:1, 1-oleoyl-2-hydroxy-sn-glycero-3-phosphate) was purchased from Avanti Polar Lipids, Inc. (Alabaster, AL) and reconstituted in 0.1% fatty acid free BSA immediately prior to use. Cisplatin, docetaxel, paclitaxel and vincristine were purchased from Sigma-Aldrich (St. Louis, MO). siRNA was purchased from Ambion (Austin, TX). RGS plasmids were purchased from the UMR cDNA Resource Center (Rolla, MO).

### Bioinformatics

Gene expression profiling data were acquired through the NCBI Gene Expression Omnibus (GEO) DataSets. The datasets GSE7556 [15], GSE15709 [16] and GSE2058 (unpublished) were downloaded and mined using Microsoft Excel prior to further analysis. Hierarchical clustering analyses was performed using Cluster developed by the Eisen lab [17] and the display of hierarchical clustering graphs utilized TreeView [17] as previously described [18]. Visual representation of the data into box plots was done with GraphPad Prism 5 (GraphPad Software, Inc., La Jolla, CA).

### RGS gene modulation

Transient transfections were performed using Fugene 6 transfection reagent (Roche Diagnostics, Basel Switzerland), according to manufacturer's instructions, at a ratio of 2 μL Fugene 6 reagent to 1 μg plasmid DNA. SKOV-3 cells were plated in 24-well plates at 50,000 cells/well and transfected with 500 ng HA-tagged RGS17 plasmid DNA, 250 ng HA-tagged RGS10 plasmid DNA, or empty vector. Assays were performed 48 hours after transient transfections, and total protein was isolated and processed for immunoblotting to confirm expression with HA epitope antibodies. For knock-down of RGS genes, SKOV-3 cells were simultaneously plated and transfected using siPORT NeoFX transfection reagent (Ambion, Austin, TX) according to the manufacturer's protocol for reverse transfection. Cells were transfected in parallel with RGS-targeted siRNA and negative control or scrambled siRNAs for each experiment. A transfection mix containing 10 nM siRNA and 2 μL siPORT NeoFX reagent in OptiMem (Invitrogen) was added to each well of a 24-well plate, followed by 30,000 cells in normal growth medium. Cells and transfection mix were incubated for 24 hours at 37°C at which point the media was changed to fresh SKOV-3 growth medium and/or cells were re-plated into either 96-well or 12-well plates. Assays were performed and samples taken for transcript expression analysis 72 h after transfection. Knock-down experiments using siRNA in HeyA8 cells and in SKOV-3 AKT assays were performed using Dharmacon ON-TARGET*plus *SMARTpools (Thermo Scientific, Lafayette, CO) and transfected using Dharmafect reagent, according to the manufacturer's recommended protocols.

### Cellular viability assays

Approximately 5,000 SKOV-3 cells were seeded in triplicates in 96-well plates in 10% FBS DMEM and allowed to attach for 24 hours prior to gene manipulation or treatment with the indicated concentrations of cisplatin, vincristine, paclitaxel or docetaxel for 48 hours. After 48 hours in the presence of chemotherapy, a cell viability assay was conducted by removing all media from the 96-well plate and replacing it with serum free media containing CellTiter-Blue^® ^reagent (Promega Corporation, Madison, WI) as previously described [19] and measured using SpectraMax M2 model microplate reader (Molecular Devices, Sunnyvale, CA).

### Quantitative real-time PCR

To determine the effect of acute cisplatin exposure on SKOV-3 cells was determined, approximately 700,000 SKOV-3 cells were plated in 100 mm tissue culture plates and allowed to attach over-night. The following day, media was aspirated and replaced with 100 μM cisplatin diluted in complete SKOV-3 media. After 24 or 48 hours of drug incubation, the media was aspirated and 7 mL Trizol reagent (Invitrogen) was added. RNA isolation was performed according to manufacturer's protocol. DNA was synthesized from 2 μg of total RNA using the High Capacity Reverse Transcriptase cDNA kit (Applied Biosystems) to amplify the mRNA. For HeyA8 parental and multi-drug resistant cells, mRNA was isolated using Trizol reagent (Invitrogen) and quantified prior to cDNA synthesis. The cDNA was synthesized using a Superscript II kit (Invitrogen) and a Mastercycler Pro (Eppendorf AG, Hamburg, Germany). Following cDNA synthesis, quantitative real-time polymerase chain reaction was performed using Superscript III kit for RT-PCR (Invitrogen) and Master Mix containing Power SYBR Green reagent (Applied Biosystems, Foster City, CA). Transcript expression was assessed using a 7900HT Real-Time PCR System from Applied Biosystems (now Life Technologies, Carlsbad, California). Reactions were normalized using the housekeeping gene GAPDH and calculations were performed according to the ΔΔCT method. Primers used were based on algorithm-generated sequences from Primer Bank (http://pga.mgh.harvard.edu/primerbank/). RGS10 Forward: GACCCAGAAGGCGTGAAAAGA, RGS10 Reverse: GCTGGACAGAAAGGTCATGTAGA, RGS17 Forward: CAGAGGAAGTCT TGTCCTGGT, RGS17 Reverse: CAAGCAAGCCAGAAAAGTAGGT, GAPDH Forward: GCCAAGGTCATCCATGACAACT, GAPDH Reverse: GAGGGGCCAT CCACAGTCTT.

### Annexin V-FITC Staining

Cells were plated and transfected with siRNA as described above and then replated in 12-well plates for ~90% confluency. Cells were treated with various concentrations of cisplatin in complete media containing 10% serum for 48 hours. The medium was then removed and cells were washed twice with PBS and incubated in binding buffer (10 mM HEPES, 140 mM NaCl, 5 mM KCl, 1 mM MgCl_2_, 1.8 mM CaCl_2_, pH = 7.4) containing annexin V-FITC (25 μg/ml) and PI (25 μg/ml) for 10 min. Cells were washed three times in binding buffer and released from the monolayers using a rubber policeman. Cells were passed through a 35 μm filter before being analyzed using a CyAN™ ADP Analyzer (Beckman Coulter, Brea, CA) to isolate and quantify annexin-positive cells, and data were analyzed using Flowjo Flow Cytometry Analysis software using established methods [20]. For each data point 20,000 cells were analyzed for SKOV-3 cells, and 50,000 cells were analyzed per data point for HeyA8 cells.

### AKT phosphorylation

SKOV-3 cells were plated in 24-well dishes at 75% confluency and transfected with Fugene 6 expression plasmid complexes or plated in 96-well dishes and transfected with siRNA complexes as described above. Cells were serum starved overnight, and treated with vehicle, 0.1 μMLPA or 1 μM LPA for five minutes at 37°C. Control wells were treated with LPA in the presence or absence of LY294002, an inhibitor of PI3 kinase. Media was then aspirated and cells were lysed in SDS-PAGE sample buffer. The lysates were boiled for five minutes and analyzed using SDS-PAGE and immunoblotting. Membranes were incubated with phospho-AKT primary antibodies (Cell Signaling Technologies), and visualized using ECL reagents (Pierce). Membranes were subsequently blotted with GAPDH antibodies as a loading control.

### Statistical analysis

Experimental data was analyzed for statistical differences using an analysis of variance (ANOVA) followed by Bonferroni's Multiple Comparison test or Tukey's test between groups, where indicated. *p < 0.05 **p < 0.01 and ***p < 0.001 indicate the levels of significance.

## Results

### Analysis of RGS expression changes in ovarian cancer models of chemoresistance

**Figure 1:**
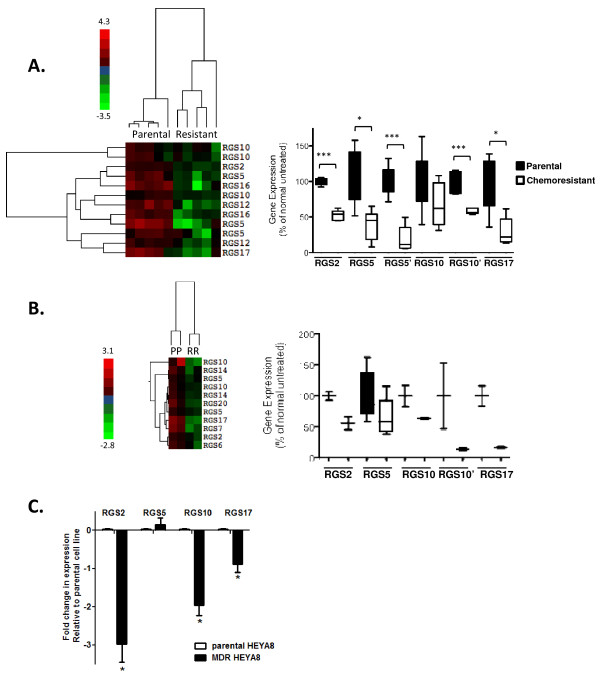
**RGS transcript expression is decreased in multiple models of ovarian cancer chemoresistance**. **(A) RGS expression is decreased in cisplatin resistant A2780 cells**. A hierarchical clustering heat map is shown depicting changes in RGS expression between parental A2780 cells (n = 5) and chemoresistant (resistant) A2780 cells (n = 5). As indicated in the side bar, red coloring represents a high level of comparative expression and green indicates a lower level (range 4.3 to -3.5). In the left panel, a box plot depicts the expression levels of multiple RGS transcripts in parental and drug resistant A2780 cells exposed to long-term cisplatin. RGS2, 5, 10, and 17 were significantly down regulated in repeated datasets and are presented here. Where multiple probes for the same gene were included on the microarray chip, these are distinguished with ('). *p < 0.05, ***p < 0.001 between groups, parental vs. chemoresistant cells. **(B) **RGS expression is decreased in vincristine-resistant SKOV-3 cells. A hierarchical clustering heat map is shown depicting changes in RGS expression between parental SKOV-3 cells (n = 2) and chemoresistant SKOV-3 cells (n = 2). In the left panel, a box plot depicts the expression levels of multiple RGS transcripts in parental (black bars) and vincristine resistant (open bars) SKOV-3 cells. **(C)** RGS expression is decreased in paclitaxel-resistant HeyA8 cells. RNA was isolated from parental and MDR-HeyA8 cells as described, and the expression of RGS2, RGS5, RGS10 and RGS17 transcripts was normalized to β2-microglobulin as an internal standard prior to the comparison between parental and multi-drug resistant HeyA8 cells. The fold change in expression relative to vehicle controls was calculated by the 2^-ΔΔCt ^method. *p < 0.05, normalized control vs. RGS groups.

We have recently demonstrated that RGS proteins significantly suppress LPA signaling in ovarian cancer cells [8,11] and that LPA mediates AKT activation and survival signals in cancer cells [8,21]. Given these connections, we explored possible roles for RGS proteins in ovarian cancer chemoresistance. To determine whether altered RGS expression correlates with acquired chemoresistance, we assessed RGS expression in multiple datasets downloaded from the NCBI Gene Expression Omnibus DataSets that contain whole-genome expression data in cultured ovarian cancer cell lines before and after acquired chemoresistance. Dataset GSE15709 describes changes in gene expression in parental chemo-sensitive A2780 cells and A2780 cells resistance to cisplatin using quintuplicate samples [16]. In the initial study, Nephew and colleagues chronically treated drug-sensitive cells with increasing concentrations of cisplatin. Following multiple rounds of clonal selection of increasingly resistant cells, they generated a multi-drug resistant cell line. Analysis of changes in RGS gene expression revealed that several RGS transcripts-RGS2, RGS3, RGS5, RGS10, RGS12, RGS16, and RGS17-were decreased in chemoresistant cells. These RGS transcripts were sufficient to distinguish between parental and resistant cell lines in hierarchical clustering analysis of expression data. To select RGS probes distinguishing chemo-resistant from parental cells, we first applied Welch's t-test to all of the probes contained within the array for RGS transcripts. The result was then visualized with a heatmap after hierarchical clustering (see Methods). This supervised approach revealed a clear correlation between decreases in selected RGS transcripts and the cisplatin-resistant phenotype (Figure 1A). We further compared the level of expression from individual RGS transcripts in parental A2780 and cisplatin-resistant cells using GSE15709 and determined that RGS2, RGS5, RGS10, and RGS17 were significantly lower in resistant cells than in parental cells (Figure 1A, ***p < 0.001, *p < 0.05). Multiple probes for RGS5 and RGS10 were present on the array, distinguished in the figure as RGS5 and RGS5', and RGS10 and RGS10'.

To confirm these results, we assessed RGS expression using an additional independent microarray gene expression dataset. In GSE7556, transcript expression in SKOV-3 cells with acquired chemoresistance to vincristine was compared to parental vincristine-sensitive cells [15]. Comparison of the changes among RGS transcripts confirmed the reduction of RGS2, RGS5, RGS10 and RGS17 in acquired chemoresistance, although the data were determined in duplicate samples, limiting the statistical significance (Figure 1B).

To further confirm that expression of these RGS transcripts is reduced in drug resistant cancer cells, we directly measured expression of RGS transcripts in parental Hey-A8 ovarian cancer cells and taxane-resistant derivatives of this cell line (commonly referred to as Multi-drug resistant or MDR-HeyA8 cells, although they retain cisplatin sensitivity; see Additional file 1). RNA was isolated from both cell lines, and transcript expression was quantified using real time RT-PCR as described in Methods. We again found that RGS2, RGS10, and RGS17 transcripts were significantly reduced in the MDR-HeyA8 cell line; however, no change was observed in RGS5 expression (Figure 1C). Taken together, these data show that RGS2, RGS10, and RGS17 transcripts are commonly downregulated in acquired chemoresistance in three distinct ovarian cancer cell lines resistant to three distinct chemotherapeutics, while RGS5 was down-regulated in two of the models. This suggests that these RGS proteins have a broad role in cell survival in the presence of multiple chemotherapeutic agents.

### Cisplatin treatment acutely lowers expression of RGS10 and RGS17 in ovarian cancer cells

**Figure 2:**
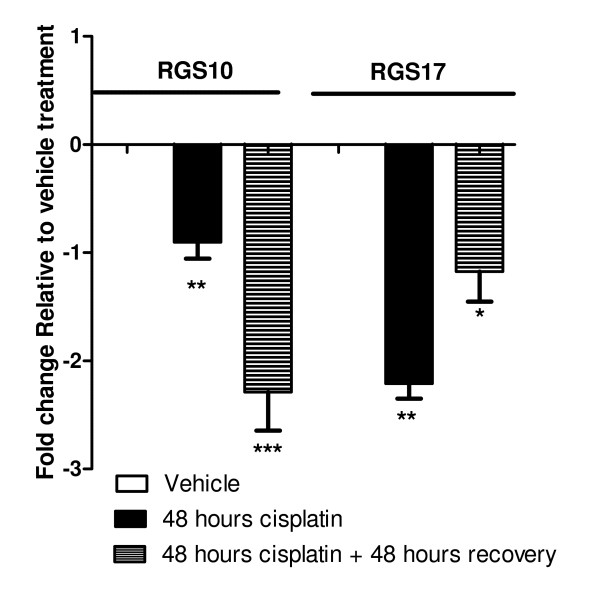
**Expression of RGS10 and RGS17 transcripts is acutely and persistently downregulated in response to cisplatin exposure**. SKOV-3 cells were treated with vehicle or the IC_80 _dose (100 μM) of cisplatin for 48 hours. RNA was isolated from the cells either immediately (black bars), or after an additional subsequent 48 hour incubation in fresh media, in the absence of drug (striped bars). The expression of RGS10 and RGS17 transcripts was normalized to GAPDH internal standards. The fold change in expression relative to vehicle controls was calculated by the 2^-ΔΔCt ^method. *p < 0.05, vehicle treated vs. cisplatin treated.

The above analysis suggests a correlation between acquired chemoresistance and decreased expression of RGS2, RGS5, RGS10 and RGS17 transcripts. Lower expression observed in chemoresistant cancer cells could reflect acutely downregulated RGS transcripts or selection for cells expressing reduced RGS transcripts. To determine if exposure to chemotherapeutics acutely causes inhibition of RGS transcripts, we treated SKOV-3 ovarian cancer cells with cisplatin for 24 and 48 hours, isolated RNA, and quantified RGS transcript expression. Cells were treated with 100 μM cisplatin, which represents approximately an IC_80 _dose, as determined by cell viability dose response curves (see Additional file 2). As shown in Figure 2, expression of RGS10 and RGS17 was reduced following a 48 h exposure to cisplatin (*p < 0.05). Significant reductions in transcript levels were also observed just 24 hours after cisplatin treatment (data not shown). To assess whether the changes were transient, we further determined the level of transcript expression after treatment with 100 μM cisplatin for 48 hours as described above, followed by removal of the drug and growth in fresh media for an additional 48 hours. Surprisingly, RGS10 and RGS17 transcript levels remained significantly lower two days following removal of the drug as compared to control cells, suggesting persistent effects on RGS expression following cisplatin exposure (Figure 2).

To determine the effects of more chronic exposure, we also treated cells with IC_30_, IC_50_, and IC_80 _doses of cisplatin for 72 and 96 hour incubations. Unfortunately, exposure times greater than 48 hours using an IC_80 _dose led to significant cell death, and treatment of cells with lower doses did not have a significant effect on RGS10 and RGS17 transcript expression (data not shown). No significant changes were observed in RGS2 or RGS5 expression following cisplatin exposure, and changes in RGS10 and RGS17 were not consistently observed following exposure to therapeutic doses of vincristine or docetaxel (data not shown).

### RGS10 and RGS17 expression levels regulate the cytotoxicity of chemotherapeutics

**Figure 3:**
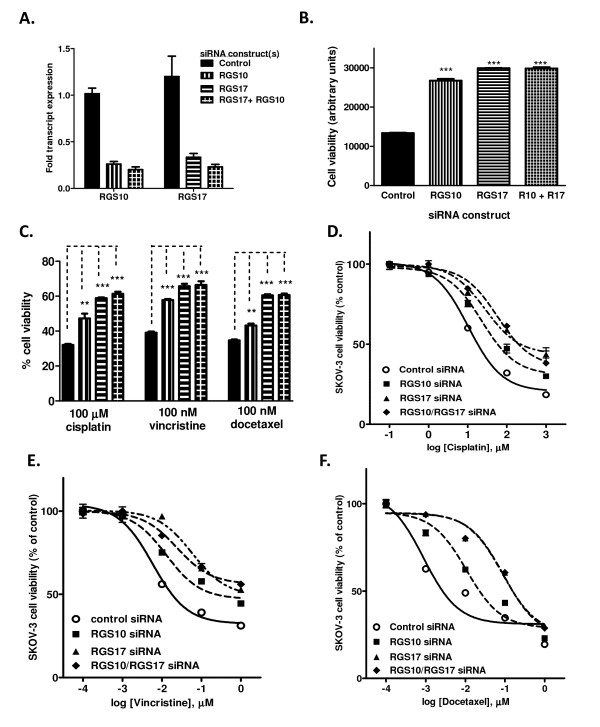
**SKOV-3 cell sensitivity to cytotoxic drugs is altered by modulating RGS10 and RGS17 expression levels**. **(A) **Transcript levels of RGS10 and RGS17 were determined using quantitative RT-PCR 72 hours after transient transfection with the indicated siRNA constructs. RGS transcript levels were normalized to GAPDH transcripts and reported relative to negative control-siRNA treated cells. **(B) **Overall cell viability was determined in SKOV-3 cells 48 hours following siRNA transfection in the absence of drug using CellTiter-Blue colorimetric cellular metabolism assays. **(C) **Cytotoxic drugs were added to cells at the indicated doses 24 hours after siRNA transfection, and cell viability was determined 48 hours after addition of drug. Data are shown normalized to cell viability in the absence of drug (100%). *: p < 0.05, **: p < 0.01,***: p < 0.001. **(D-F) **siRNA-mediated knock-down of RGS10 or RGS17 alone or in combination resulted in a right-shifted dose response curve of cisplatin **(D)**, vincristine **(E)**, or docetaxel **(F) **toxicity in SKOV-3 cells.

We next determined if directly inhibiting RGS expression could recapitulate the observed loss of chemosensitivity. For the following experiments, we focused on RGS10 and RGS17 because they were downregulated in three independent models of chemoresistance, and, unlike RGS2, they selectively deactivate Gi family G-proteins [22-24], which are known to regulate survival pathways [8,25,26]. To determine if the loss of RGS10 and/or RGS17 expression could be directly linked to a change in sensitivity to chemotherapeutics, we determined the effect of siRNA mediated knock-down of RGS10 and/or RGS17 on cell viability in the absence or presence of chemotherapeutics. Transfection of siRNA duplexes targeted at RGS10 and RGS17 resulted in 75-85% reduction of each respective transcript whether transfected alone or in combination (Figure 3A). We first assessed changes in cell viability mediated by changes in RGS expression levels in the absence of any chemotherapeutic drug. Reduced expression of either RGS10 or RGS17 resulted in significantly higher cell viability 72 hours after siRNA transfection (Figure 3B).

We further assessed the ability of RGS10 and RGS17 levels to affect cell death induced by three cytotoxic chemotherapy agents: the platinum compound cisplatin, the taxane compound docetaxel, and vincristine. Platinum and taxane compounds are used in first-line chemotherapy regimens in ovarian cancer (paclitaxel usage in ovarian cancer is an FDA-approved labeled indication while docetaxel is off-label for this indication). Vincristine is used for other tumor types (acute lymphoblastic leukemia, Hodgkin's lymphoma, multiple myeloma, etc.), but was included in the analysis to determine how broad the effects of RGS proteins were. SKOV-3 cells display low sensitivity to cisplatin, requiring micromolar doses for significant cell death. In contrast, these cells are highly sensitive to vincristine and docetaxel (see Additional file 2). Cell viability was determined following 48 hour treatment with 100 μM cisplatin, 100 nM vincristine, or 100 nM docetaxel in cells transfected with either negative control siRNA, RGS10 siRNA, RGS17 siRNA or both RGS siRNA constructs. The percent cell viability (normalized to cell number in the absence of drug, to account for the effect on overall cell growth as shown above) was significantly higher in cells with reduced RGS10 or RGS17 expression levels (Figure 3C). Dose response curves show that lowering either RGS10 or RGS17 transcript levels resulted in a decrease in the potency of cisplatin, vincristine, and docetaxel (Figure 3D-F, Table 1). Simultaneous knock-down of RGS10 and RGS17 did not markedly enhance the effect of individual knock-downs. These data suggest that ovarian cancer cells may have survival signals which are normally blunted by endogenous RGS10 and RGS17 expression. Notably, the fold decrease in RGS10 and RGS17 transcript expression achieved following siRNA treatment that led to this increase in cell survival is comparable to that observed following cisplatin exposure, indicating that cisplatin exposure itself may reduce the sensitivity of SKOV-3 cells to cisplatin by inhibiting RGS10 and RGS17 expression.

**Table 1:** Potencies of chemotherapeutics following siRNA treatment in SKOV-3 cells.

	Scrambled siRNA	RGS10 siRNA	RGS17 siRNA	RGS10 + RGS17 siRNA
**Vincristine Potency (nM IC**_**50 **_**+/- SEM)**	5. 85 +/- 1.3	12.8 +/- 2.9	58 +/- 8.7	24 +/- 4.8
**Docetaxel Potency (nM IC**_**50 **_**+/- SEM)**	0.886 +/- 0.432	10.7 +/- 3.7	85 +/- 23	92 +/- 25
**Cisplatin Potency (μM IC**_**50 **_**+/- SEM)**	10.4 +/- 1.1	23.0 +/- 1.2	27.9 +/- 1.2	57.8 +/- 1.5

We next performed the reciprocal experiment by overexpressing either RGS10 or RGS17 in SKOV-3 cells, with the expectation that this may increase the potency of cisplatin-induced cell death. Indeed, in some experiments RGS10 and RGS17 overexpression did enhance chemotherapeutic potency, but the effect was not consistent, varying with transfection efficiency (data not shown). It is possible that the endogenous levels of RGS10 and RGS17 are sufficiently high to provide near-maximal GAP activity, such that only extremely high levels of transient transfection produce a significant change in activity levels. Nonetheless, the decrease in cytotoxicity of chemotherapeutics following RGS10 or RGS17 knock-down clearly indicate that the suppression of these proteins promotes cell survival and suggest a decrease in the expression levels of RGS10 or RGS17 are sufficient to lower ovarian cancer cell sensitivity to chemotherapeutic cytotoxicity.

### RGS10 and RGS17 regulate chemotherapeutic-induced cell death

**Figure 4:**
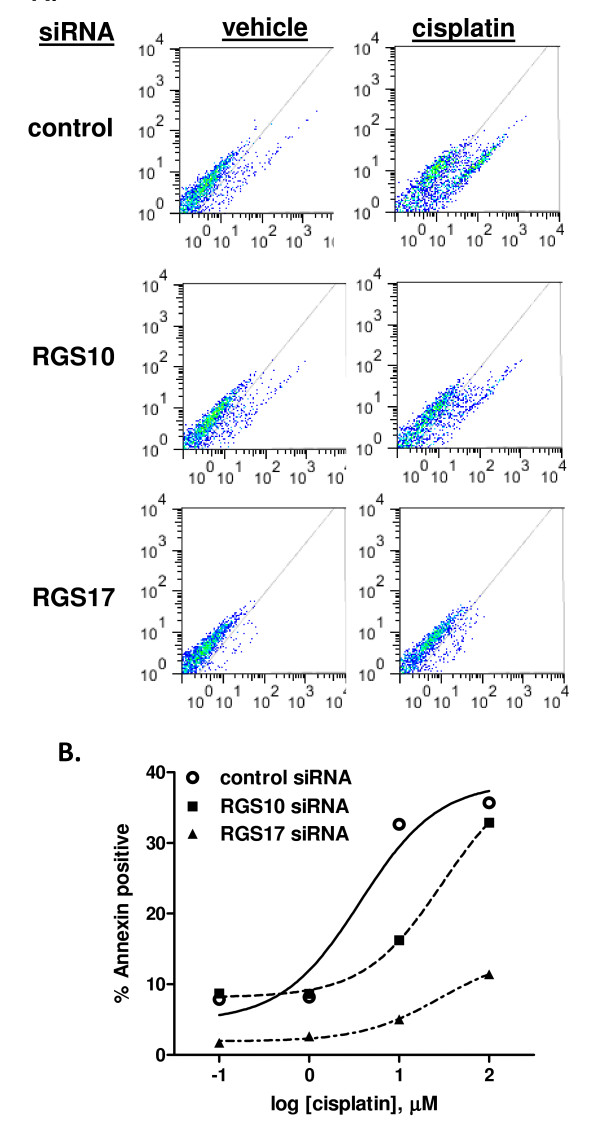
**RGS10 and RGS17 knock-down suppresses cisplatin-induced cell death**. **(A) **SKOV-3 cells were transiently transfected with control or RGS-targeted siRNA constructs and treated with vehicle or 10 μM cisplatin for 48 hours. Cells were then fixed and stained with annexin V-FITC to quantify externalized phosphatidyl serine. Cells were analyzed by flow cytometry, and gated to calculate percentage of cells staining positively for annexin. In each plot, PI staining intensity is indicated on the y-axis and annexin staining intensity is indicated on the x-axis. Cells to the bottom and right of the indicated diagonal line were scored as annexin positive. **(B) **The percentage of annexin-positive cells was determined at each siRNA transfection condition in the presence of increasing doses of cisplatin. 20,000 cells were analyzed for each data point.

The data above demonstrate that RGS10 and RGS17 expression alters sensitivity to chemotherapeutic-induced cytotoxicity when assessed by viability assays, which suggests a decrease in cell death. To more specifically evaluate the role of RGS proteins in regulating cell death stimulated by chemotherapeutics, we assessed chemotherapeutic induced phosphatidyl-serine (PS) externalization, a hallmark of cell death. SKOV-3 cells were transfected with either negative control (NC), RGS10 or RGS17 siRNA, and then exposed to vehicle, 1 μM, 10 μM, or 100 μM cisplatin for 48 hours. PS externalization was assessed using annexin staining and flow cytometry as described [20]; 20,000 cells were counted for each data point. We found that cells with reduced RGS10 or RGS17 expression displayed less cisplatin-induced cell surface annexin staining (Figure 4). Notably, both apoptotic and non-apoptotic mechanisms of cell death may contribute to cisplatin toxicity, and measuring PS externalization at this time point accounts for cell death by either mechanism. This suggests that lowering RGS expression levels blunts the ability of cisplatin to induce cell death in SKOV-3 ovarian cancer cells. We also determined the effect of RGS10 knock-down on cell proliferation by assessing BrdU incorporation (data not shown). We did not observe a significant effect on the rates of cell proliferation, suggesting that the observed effects of RGS knock down on cell number and cell viability reflect a decrease in cell death rather than an increase in cell proliferation.

#### MDR-HeyA8 sensitivity to cisplatin cytotoxicity is regulated by RGS10 expression

**Figure 5:**
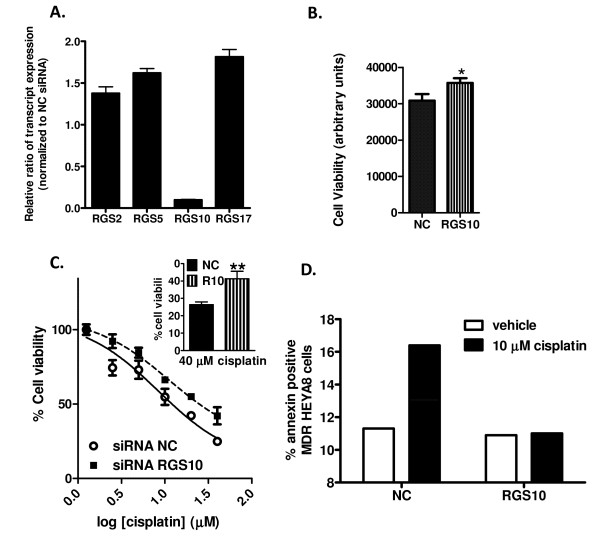
**RGS10 alters cytotoxicity of cisplatin in multi-drug resistant HeyA8 cells**. **(A) **RT-PCR indicates selective knock-down of RGS10 48 hours following transfection of MDR-HeyA8 cells with RGS10 siRNA. **(B) **siRNA mediated knock-down of RGS10 results in increased cell growth as determined by viability assays. *: p < 0.05 **(C) **Cisplatin was added at the indicated doses 24 hours after siRNA transfection, and cell viability was determined 48 hours after drug treatment. Data are shown normalized to cell viability in the absence of drug (100%). Inset: Relative cell viability of RGS10 siRNA transfected MDR-HeyA8 cells is significantly higher than control transfected cells in the presence of 40 μM cisplatin. **: p < 0.01. **(D) **Phosphatidylserine externalization was determined by annexin staining in MDR-HeyA8 cells transfected with control or RGS10 siRNA following 48 hour incubation with vehicle or 10 μM cisplatin. Annexin staining was determined using flow cytometry as described in Methods. 50,000 cells were analyzed for each data point.

To confirm that our observation that direct suppression of RGS expression by siRNA decreases sensitivity to chemotherapeutic drug induced cytotoxicity was not specific to SKOV-3 cells, we also determined the effect of RGS knock-down on cell viability, cell proliferation, and cell death in Hey-A8 cells. RGS10 siRNA resulted in selective loss of RGS 10 transcript (Figure 5A). However, in our hands RGS17 siRNA resulted in non-selective knock-down in HeyA8 cells, unlike the selective effects seen in SKOV-3 cells. Thus, we report here the effects of only RGS10 siRNA. siRNA transfection resulted in approximately 80% knock-down of RGS10 transcript MDR-HeyA8 cells and the effects on cell viability and death were strikingly similar to those observed in SKOV-3 cells. Knock-down of RGS10 transcript resulted in a small but significant and reproducible increase in cell viability (Figure 5B). Further, the relative cell viability in the presence of micromolar doses of cisplatin (normalized to that seen in the absence of drug) was significantly higher with RGS10 siRNA knock-down (Figure 5C). Also similar to results in SKOV-3 cells, we confirmed that RGS10 knockdown blunts cell death stimulated by cisplatin (Figure 5D), while it has no effect on cell proliferation, as measured by BrdU incorporation (data not shown). Thus, RGS10 knock-down has similar effects in SKOV-3 cells and MDR-HeyA8 cells. Surprisingly, while siRNA transfection resulted in knock-down of RGS10 transcript in parental HeyA8 cells as well, this knock-down did not consistently alter cell viability in the presence of cisplatin (discussed below).

### RGS10 and RGS17 suppress AKT activation

**Figure 6:**
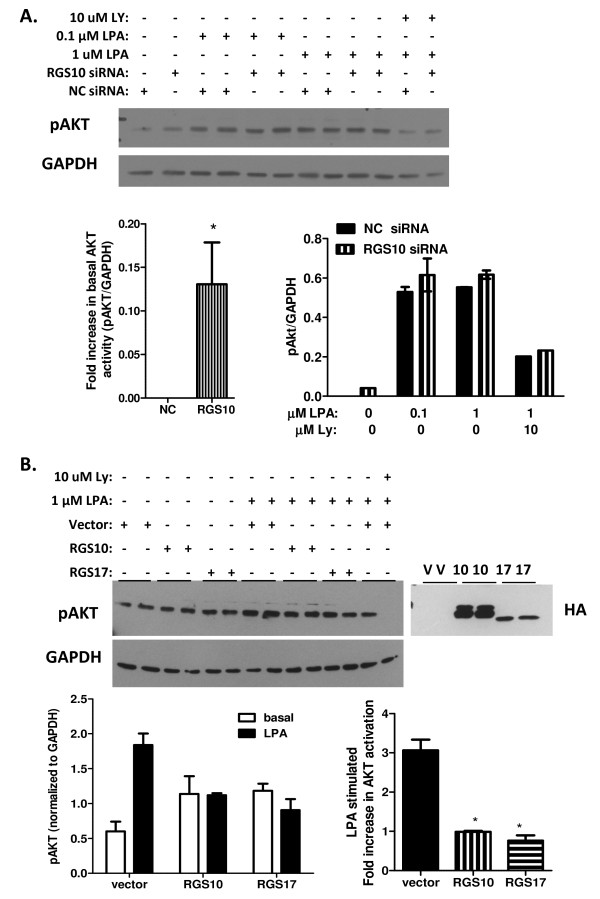
**RGS and RGS17 inhibit AKT activation**. **(A) **SKOV-3 cells transfected with control (NC) or RGS10 (10) siRNA constructs were serum starved and stimulated with vehicle or LPA at the indicated concentrations for 5 minutes. Control wells were pre-incubated with LY294002, a PI3K inhibitor, for 1 hour. Cell lysates were analyzed with SDS-PAGE and immunoblotting with anti-phospho AKT antibodies (top) to measure AKT activation. Phospho-AKT blots were re-probed with GAPDH antibodies as loading controls (bottom). Activation of AKT was quantified by densitometry of bands and normalized to GAPDH controls. **(B) **SKOV-3 cells were transfected with either empty vector (V), plasmid encoding HA-tagged RGS10 (10), or plasmid encoding HA-tagged RGS17 (17). Cells were serum-starved overnight and treated with vehicle or LPA for 5 minutes at 37°C. Control cells were also treated with 10 μM LY294002 (LY) to block activation. Cell lysates were analyzed with SDS-PAGE and immunoblotting with anti-phospho AKT antibodies (top left) to measure AKT activation, or HA antibodies (top right) to confirm transfection. Phospho-AKT blots were re-probed with GAPDH antibodies as loading controls (bottom). Activation of AKT was quantified by densitometry of bands and normalized to GAPDH controls.

Our observation that inhibition of RGS10 and RGS17 expression enhances cell survival in the presence of chemotherapeutics suggests that endogenous RGS10 and RGS17 may suppress constitutive survival signals in ovarian cancer cells. An obvious candidate survival pathway is the PI3K/AKT pathway, which is activated in ovarian cancer cells via Gi G-proteins, targets of deactivation by RGS10 and RGS17 [8]. Further, LPA is a known survival factor for ovarian cancer cells, and it is constitutively generated and released from ovarian cancer cells, resulting in autocrine activation of AKT pathways [8,27]. Given that RGS10 and RGS17 selectively deactivate Gi subunits [23,28], we hypothesized that RGS10 and RGS17 may inhibit LPA-stimulated AKT activation. To test this possibility, we serum starved SKOV-3 cells transfected with control or RGS10 siRNA to remove serum-bound LPA, and then measured basal and acute LPA-stimulated AKT phosphorylation. We did not observe a difference in the ability of LPA to stimulate AKT phosphorylation between cells expressing endogenous levels of RGS10 and cells with 75-80% knock-down of RGS10. However, basal AKT phosphorylation levels were slightly but consistently and significantly higher in cells with reduced RGS expression, suggesting that endogenous RGS10 expression levels may function to attenuate AKT-mediated survival signaling (Figure 6A). It is possible that endogenous levels of RGS proteins are sufficiently high in SKOV-3 cells that LPA stimulated AKT levels may not be affected after siRNA knock-down of RGS10, even if RGS10 proteins are capable of negatively regulating LPA stimulated AKT signaling. To determine if RGS10 and RGS17 are capable of regulating this effect, we measured LPA-stimulated AKT activation in SKOV-3 cells expressing basal levels of RGS proteins and cells overexpressing each isoform. The ability of LPA to stimulate AKT phosphorylation was markedly blunted in cells overexpressing either RGS protein (Figure 6B), suggesting that RGS10 and RGS17 proteins may deactivate Gi subunits required for LPA stimulated AKT activation in SKOV-3 cells.

## Discussion

**Figure 7:**
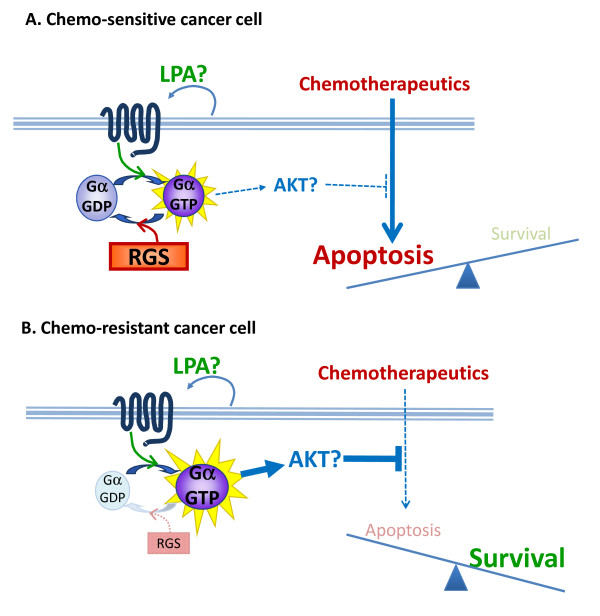
**Model of working hypothesis**. We hypothesize that suppression of RGS expression contributes to chemoresistance by indirectly amplifying autocrine survival signals.

Chemoresistance is a significant problem in ovarian cancer and prevents a cure until it can be better understood scientifically and then managed clinically. Refractory tumors have poorer outcomes and require innovative strategies to re-sensitize tumors to chemotherapy and/or require the development of strategic therapeutics that will bypass chemoresistance. This study introduces RGS10 and RGS17 as novel mediators of chemoresistance in ovarian cancer cells. After our initial observations of a correlation between low levels of RGS transcript expression in acquired chemoresistance, we established a causative relationship between suppression of RGS10 and RGS17 and a reduced susceptibility to chemotherapeutic cytotoxicity. Further, RGS10 and RGS17 suppress activation of the survival factor AKT. Based on these data, we propose that suppressed expression of RGS proteins is part of the molecular mechanism that allows tumorigenic cells to resist chemotherapy. Our working model of this mechanism suggests that autocrine or paracrine activation of Gi-coupled receptors such as LPA receptors activates survival pathways such as those mediated by AKT that oppose chemotherapy-induced cell death. Thus, we hypothesize that RGS10 and RGS17 function as tumor suppressors by blunting endogenous survival pathways. The level of expression of endogenous RGS proteins critically determines whether the balance shifts towards apoptosis or survival (Figure 7).

The current study focuses on the role of RGS10 and RGS17 in determining chemoresistance in ovarian cancer cells. There are over 20 genes encoding mammalian RGS proteins, and over 30 distinct gene products, ranging from small proteins comprised solely of an RGS domain which mediates GAP activity, to multi-domain proteins with multiple targeting and regulatory functions [29,30]. RGS proteins critically regulate the strength and duration of G-protein mediated signals by accelerating the rate by which activated GTP-bound Gα subunits are deactivated and returned to the inactive GDP-bound form up to 1000-fold [31]. As a result, RGS proteins are critical to regulating the kinetics and amplitude of physiological signal transduction cascades. Indeed, profound physiological effects have been observed in visual and cardiovascular signaling systems upon loss of RGS expression [32,33].

RGS10 and RGS17 are among the smallest of the RGS proteins, lacking well defined functional domains outside of the RGS domain. RGS17 is a member of the RZ (A) subfamily of RGS proteins and preferentially deactivates members of the Gi/o family of G-proteins [28,34]. RGS17 contains a palmitoylation site N-terminal of the RGS domain, which potentially regulates its subcellular localization, further impacting G-protein and receptor selectivity. Clinical expression data available through the Oncomine database suggests that RGS17 is expressed at significantly lower levels in ovarian tumors compared to normal ovary, with expression of RGS17 progressively decreasing with advancing disease grade [13]. This trend is consistent with our observation that RGS17 expression is lost during chemoresistance, although the chemosensitivity of the tumors in these datasets was not reported. Notably, our results are in contrast with a recent study showing that RGS17, which is overexpressed in human lung and prostate cancer [35-37], induces tumor cell proliferation [38]. In lung cancer cells, RGS17 proliferative effects are mediated by cAMP activation of PKA and CREB. We did not observe any proliferative effects of RGS17 in our system, and it is unknown if RGS17 can simultaneously activate cAMP/proliferative pathways and inhibit AKT/survival pathways. Further work will determine if this is a cell-type specific pathway.

RGS10 is a member of the R12 (D) subfamily of RGS proteins, although RGS10 lacks the multiple regulatory domains found in other R12 family members. RGS10 has sites for regulatory palmitoylation and phosphorylation which have been shown to regulate its subcellular localization. Like RGS17, RGS10 GAP activity is selective for Gi/o family G-proteins [23]. Changes in RGS10 expression in ovarian cancer have not been reported, but interestingly it has recently been shown that black tea polyphenols, which induce apoptosis in human colon cancer cells, cause acute upregulation of RGS10 expression [39]. Our findings that RGS10 negatively regulates survival signals suggest that the upregulation of RGS10 may be linked to polyphenol-induced apoptosis. While not a major focus of our study, we did observe consistent down-regulation of RGS2 in chemoresistant ovarian cancer cells. This is consistent with a report by Tu and colleagues that RGS2 is down-regulated in androgen-independent prostate cancer cells, and RGS2 expression suppresses growth in those cells [40].

Our initial findings are based on analysis of previously reported datasets describing whole-genome expression changes during acquired chemoresistance in ovarian cancer [15,16]. In addition to the changes in RGS transcripts, on which we specifically focused, the original report of transcriptional changes in vincristine-resistant SKOV-3 cells identified changes in a wide array of genes associated with chemoresistance. Strikingly, there was significant overlap between these chemoresistance-related transcripts and our previously published LPA-induced transcriptome, transcripts that we identified as being specifically regulated following LPA treatment in OVCAR-3 and SKOV-3 cells [15,18]. LPA mediates its effects by activating a family of GPCRs, which each couple to heterotrimeric G-proteins and stimulate activation of the G-protein upon LPA binding to the receptor. It is the activated G-protein-the target for RGS deactivation - that mediates the cellular effects of LPA, including growth, migration, and of particular interest here, survival. Taken together, these observations suggest convergence between transcriptional changes that mediate chemoresistance and activation of pathways downstream of LPA.

While there is circumstantial evidence for a role for LPA survival pathways in the observed link between RGS proteins and chemoresistance, there are inconsistencies that suggest LPA is at least not the whole story. For example, we observed that the overexpression of RGS10 and RGS17 suppressed LPA-stimulated AKT phosphorylation, while overexpression had inconsistent effects on cisplatin induced cell death. Conversely, RGS10 knock-down had significant effects on cell viability, but did not significantly affect LPA stimulated AKT activation. Several potential differences in the experimental conditions could account for this, for example viability assays to determine cisplatin-induced cellular toxicity were performed 72 hours following transfection in the presence of chemotherapeutic drug, while LPA-stimulated AKT phosphorylation was measured 48 hours after transfection following serum starvation and acute 5 minute LPA treatment. It is also possible that the endogenous expression levels of RGS are sufficient to maximally deactivate G-proteins under basal, autocrine LPA stimulating conditions, but following acute LPA receptor activation, deactivation of G-proteins by endogenous RGS proteins cannot keep up the with activation of G-proteins by LPA receptors, making the effect of exogenous RGS proteins more apparent. However, other Gi-coupled GPCRs are implicated in ovarian cancer proliferation and survival signaling, such as endothelin, chemokine, and prostaglandin receptors [41-43], suggesting RGS10 and RGS17 may regulate multiple survival pathways besides LPA.

Similar effects on cell viability and cell death were seen in SKOV-3 and MDR-HeyA8 cells, which both show low sensitivity or resistance to certain drugs, but the effects were not recapitulated in parental HeyA8 cells. It is possible that additional molecular changes have occurred in the resistant cells that facilitates survival in the presence of reduced RGS knock-down. It is also possible that parental HeyA8 cells have sufficiently high levels of RGS10 expression that knock-down does not change the rate of G-protein deactivation. In contrast, a similar fold knock-down in MDR-HeyA8 cells, which we shown have several fold lower RGS10 expression than parental cells, may be sufficient to change the kinetics of G-protein deactivation. Future research is required to define the thresholds below which RGS expression becomes rate limiting and how this survival mechanism interacts with other survival adaptations facilitating chemoresistance.

## Conclusions

Although medical science is on the verge of developing novel, selective anti-cancer therapeutics with tumor-specific targets, "traditional" cytotoxic chemotherapeutic agents such as platinum compounds and taxanes still remain a dominant force in the fight against cancer and are highly effective against specific tumor types. It is critical to understand the molecular changes that lead to chemoresistance in order to predict, prevent, and reverse refractory disease, thereby resurrecting the therapeutic potential of the available arsenal of effective chemotherapies. Our results establish RGS10 and RGS17 as novel regulators of cell survival and chemoresistance in ovarian cancer cells and represents the first link between any RGS protein and cancer chemoresistance. Our findings are critical to understanding the transitional changes that tumor cells undergo along the path to chemoresistance. Establishing this important mechanism involving RGS10 and RGS17 is the first part of a systematic evaluation of cellular signaling in ovarian cancer chemoresistance.

## Competing interests

The authors declare that they have no competing interests.

## Authors' contributions

SBH designed and analyzed data for all experiments except bioinformatics and co-wrote the manuscript. MMM designed and analyzed data for experiments, including bioinformatics data and co-wrote the manuscript. CPC performed annexin experiments and AKT siRNA experiments. MKA performed HeyA8 viability and RT-PCR experiments and assisted with SKOV-3 viability experiments. MWA performed AKT overexpression assays and RGS downregulation assays. JHH assisted with siRNA and overexpression experiments, annexin staining and RGS down regulation. All authors read and approved the final manuscript.

## Supplementary Material

Additional file 1 **Drug sensitivity of parental HeyA8 cells and multi-drug resistant (MDR) HeyA8 cells**. The viability of each cell line was determined following 48 hour treatment with various concentrations of each drug using Cell Titer Blue metabolic viability assays as described. Click here for file

Additional file 2 **Drug sensitivity of SKOV-3 cells**. The viability of SKOV-3 cells was determined following 48 hours treatment with various concentrations of each drug using Cell Titer Blue metabolic viability assays as described. Click here for file
