# Supplementary figures and images for: Regulators of G-Protein signaling RGS10 and RGS17 regulate chemoresistance in ovarian cancer cells
Source: Mol Cancer. 2010 Nov 2;9:289. doi: 10.1186/1476-4598-9-289 (PMC2988731; doi:10.1186/1476-4598-9-289)

## Slide 1
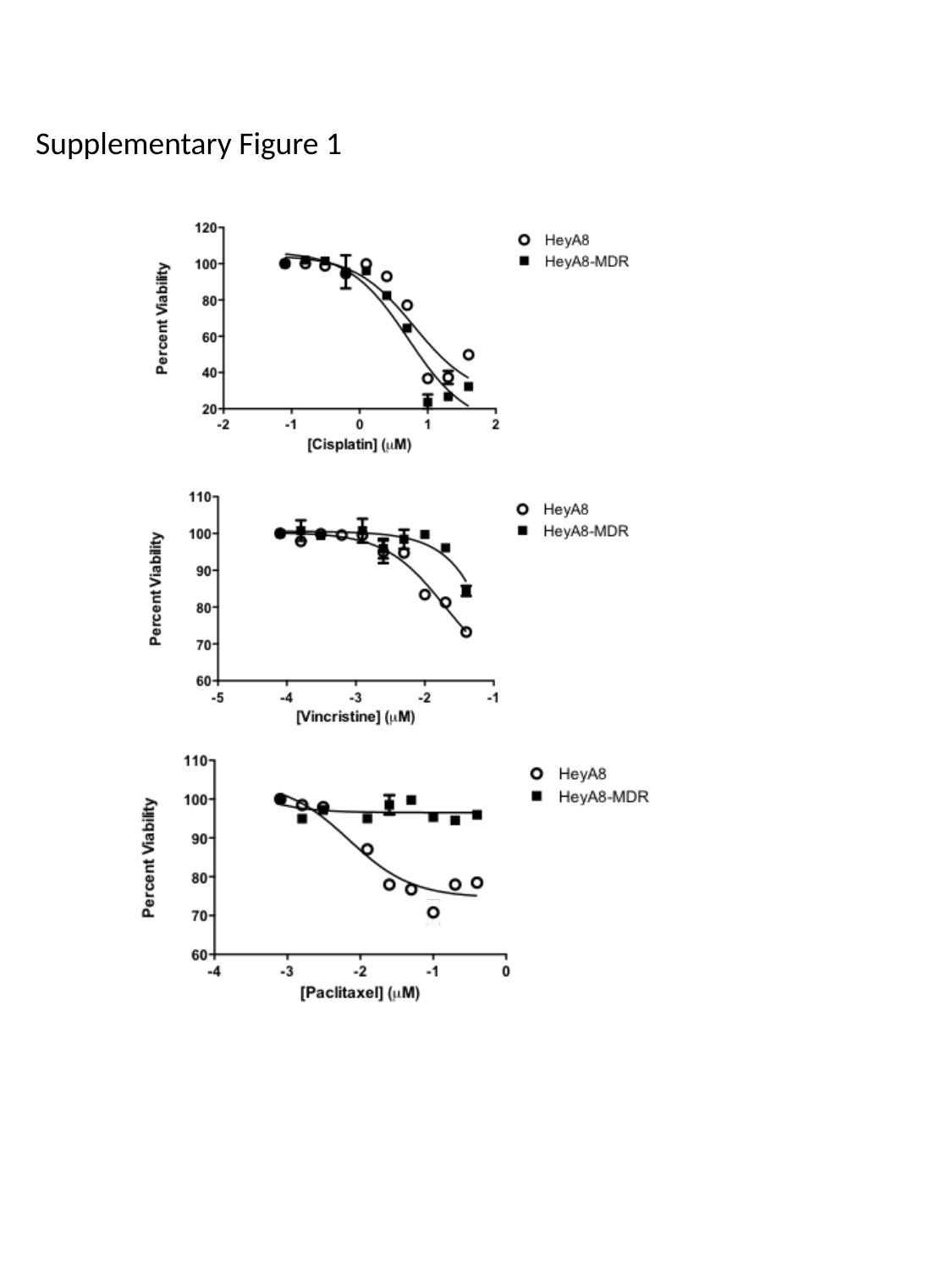

Supplementary Figure 1

Supplement: Additional file 1 — Drug sensitivity of parental HeyA8 cells and multi-drug resistant (MDR) HeyA8 cells. The viability of each cell line was determined following 48 hour treatment with various concentrations of each drug using Cell Titer Blue metabolic viability assays as described. [file 1476-4598-9-289-S1.PPT]

## Slide 1
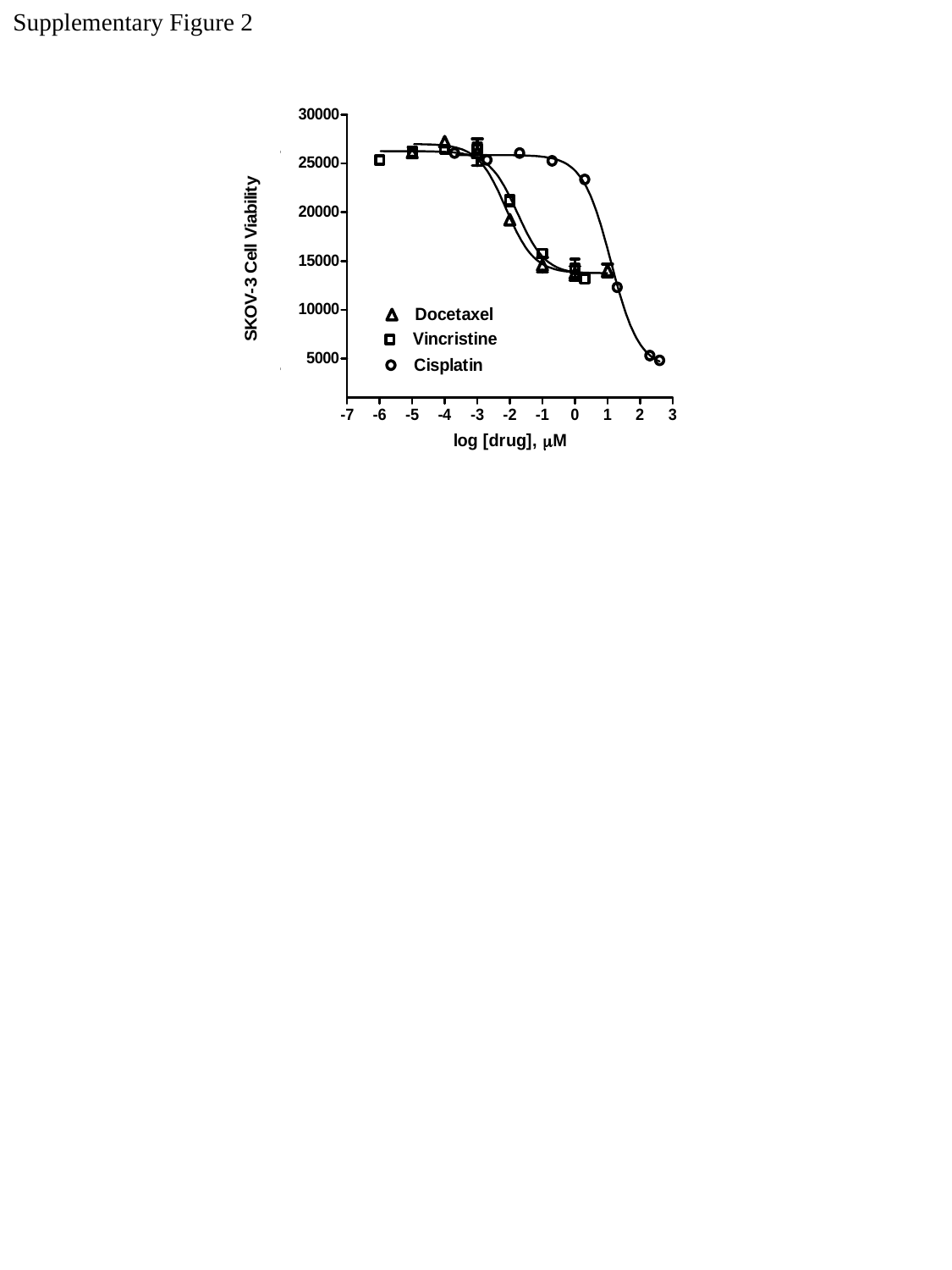

Supplementary Figure 2

Supplement: Additional file 2 — Drug sensitivity of SKOV-3 cells. The viability of SKOV-3 cells was determined following 48 hours treatment with various concentrations of each drug using Cell Titer Blue metabolic viability assays as described. [file 1476-4598-9-289-S2.PPT]
